# Hygiene Knowledge and Digital Health Literacy in Emergency Medical Services in Germany: A Cross-Sectional Study of EMS Personnel and Emergency Physicians with Relevance for the Development of Training Programmes

**DOI:** 10.3390/healthcare14183091

**Published:** 2026-09-19

**Authors:** Veit Kinne, Dragisa Mitic, Nora Helke Leder, Sebastian Lang, Kristin Büchel, Frank Kipp, Sabine Trommer

**Affiliations:** 1 Institute for Infectious Diseases and Infection Control, Jena University Hospital, 07747 Jena, Germany; 2 Department of Anaesthesiology and Intensive Care Medicine, Jena University Hospital, 07747 Jena, Germany; 3 Fire Service Department of the City of Jena, 07743 Jena, Germany

**Keywords:** emergency medical services, infection prevention, hand hygiene, personal protective equipment, multidrug-resistant organisms, digital health literacy, patient safety, teamwork

## Abstract

**Highlights:**

**What are the main findings?**

- WHO-based hand hygiene knowledge among EMS personnel and emergency physicians was generally moderate to good, whereas the extended hygiene knowledge test on basic hygiene measures, PPE use, and MDRO management indicated knowledge gaps in additional areas.
- Digital health literacy differed between EMS personnel and emergency physicians, particularly in dimensions related to information searching, evaluating reliability, determining relevance, and protecting privacy.

**What are the implications of the main findings?**

- The findings may support the development and implementation of targeted hygiene and infection prevention training for EMS personnel, with particular attention to PPE use, MDRO management, and indication-based decision-making.
- Future training and evaluation approaches should go beyond knowledge assessment and include additional learning dimensions, such as practical skills, clinical decision-making, attitudes, and observed hygiene behaviour.

**Abstract:**

**Background:** Effective hygiene and infection prevention are essential for patient and staff safety in EMS. This study assessed hygiene and infection prevention knowledge among EMS personnel and emergency physicians in Thuringia, Germany. Specifically, it examined knowledge of WHO recommendations on hand hygiene, standard infection prevention measures, appropriate use of personal protective equipment (PPE), and the management of patients with confirmed multidrug-resistant organisms (MDROs). In addition, associations with digital health literacy, job satisfaction, teamwork, and patient safety were explored to inform future training needs in EMS. **Methods:** A quantitative cross-sectional study was conducted using an online questionnaire between June and September 2025. A total of 72 EMS personnel and emergency physicians participated. The survey included the Digital Health Literacy Instrument (DHLI), the WHO questionnaire on hand hygiene knowledge, a knowledge test on standard infection prevention measures, PPE use, and MDRO management, the short questionnaire on general and facet-specific job satisfaction (KAFA), and the German version of the questionnaire on teamwork and patient safety (FTPS/SAQ). Group comparisons were performed using Fisher’s exact test or *t*-tests, as appropriate, and associations were examined using correlation analyses. **Results:** WHO-based hand hygiene knowledge was predominantly moderate (59.5%). Emergency physicians demonstrated higher levels of knowledge than EMS personnel (57.1% vs. 28.6%) and were more likely to answer basic questions on transmission routes correctly. In the extended hygiene knowledge test on basic hygiene measures, PPE use, and MDRO management, most participants showed low (43.1%) or moderate knowledge (45.1%), while only 11.8% achieved a good level. Digital health literacy was generally high, particularly for basic application skills. Respondents reported a high level of job satisfaction. Teamwork and patient safety were also perceived positively overall; however, communication was rated less positively by emergency physicians than by EMS personnel (*p* = 0.005; d = 0.81). **Conclusions:** Relevant gaps in hygiene and infection prevention knowledge were identified in the EMS setting, particularly regarding standard infection prevention measures, PPE use, and MDRO management. Future EMS training should therefore address both knowledge gaps and implementation-relevant factors such as communication, teamwork, and digital competencies. This study should be regarded as an exploratory pilot study.

## 1. Introduction

Hand hygiene is the cornerstone of infection prevention and is widely recognised as the most effective and cost-effective measure to reduce healthcare-associated infections (HAIs) [1], thereby representing a key component of patient safety [2]. Hand hygiene training programmes are broadly integrated into curricula for physicians, nurses, and EMS personnel. Nevertheless, HAIs remain a substantial challenge to patient safety [3]. According to recent estimates from the European Centre for Disease Prevention and Control (ECDC), approximately 4.3 million patients in the European Union (EU) and European Economic Area (EEA) acquire at least one HAI each year, corresponding to about 4.8 million HAI episodes annually [3]. HAIs, which may occur as a result of inadequate hygiene practices, are associated with increased mortality, a substantial economic burden, and considerable patient suffering [4]. 

EMS personnel encounter a large number of patients in diverse operational environments and may therefore contribute to the transmission of pathogens. Prehospital EMS personnel are regularly exposed to potentially infectious agents. The often confined working environment of EMS personnel, such as ambulances or private homes, facilitates the transmission of infectious agents to patients, colleagues, and indirectly to the healthcare system as a whole. The systematic review by Obenza et al. [5] demonstrated that pathogens associated with healthcare-associated infections (HAIs) were frequently detected on a variety of surfaces in the ambulance patient care area, including blood pressure cuffs, oxygen equipment, and components of patient stretchers such as handles, side rails, and headrests. 

The implementation of and adherence to hygienic hand disinfection, one of the most effective measures for preventing nosocomial infections, has rarely been studied in EMS to date. Vikke et al. [4] examined 1344 indications for hand disinfection among 77 paramedics and found an overall compliance rate of only 15%. Compliance was critically low before patient contact (3%) and before aseptic procedures (2%), while it increased after contact (after patient contact 29%, after patient environment 38%). This asymmetry suggests that EMS personnel prioritise self-protection and increasingly use gloves (54% of all indications), often without an evidence-based rationale [4]. 

The review by Alsaleem et al. [6] also showed that compliance with infection control measures by EMS personnel is inadequate. The barriers to the implementation of infection control measures in EMS identified in the study by Alsaleem et al. [6] are complex and span individual, organisational, and operational levels. In addition to frequent underestimation of the risk of infection and staff fatigue, time pressure, unpredictable operational conditions, and insufficient training and availability of protective equipment make it particularly difficult to consistently comply with hygiene standards [6]. 

A recent study by Duwal et al. [7] demonstrated that compliance with hand hygiene measures among the healthcare professionals surveyed (physicians, nurses, EMS personnel) was only 30% overall. Physicians had the highest hand hygiene compliance (37%), followed by nurses (35%) and EMS personnel (23%). Overall, these findings from Duwal et al. [7] illustrate that hand hygiene measures continue to be inadequately implemented in all occupational groups. 

Bucher et al. [8] reported poor overall hand hygiene among EMS personnel, identifying significant deficiencies in various clinical situations, particularly before patient contact, after invasive procedures, and after use of personal protective equipment (PPE). PPE is also often used incorrectly or incompletely. Northington et al. [9] demonstrated that only 14.3% of EMS personnel were capable of donning and doffing PPE without making critical errors. The most common errors were the premature removal of the respirator (65.7%) and contact between the contaminated suit and the body (54.3%) [9]. The most common non-critical error observed was potential self-contamination due to boots not being removed before other parts of the body were exposed (37.1%) [9]. This highlights the need for future interventions to improve hand hygiene practices, promote the proper use of PPE, and implement basic hygiene measures in EMS. 

In EMS, paramedics and emergency physicians frequently operate in constantly changing environments and under considerable time pressure. This makes the consistent implementation of hygiene and infection prevention measures particularly challenging. Effective prevention requires expertise in areas such as hand hygiene, PPE, and the management of multidrug-resistant organisms (MDROs). It also requires digital skills to access and apply evidence-based information, as well as awareness of contextual factors such as teamwork, patient safety culture, and job satisfaction. Digital health literacy may therefore support paramedics and emergency physicians in accessing, evaluating, and applying digital health information. 

Prior to implementing a blended learning strategy involving train-the-trainer sessions and Moodle-based training, it is necessary to systematically assess hygiene knowledge and potentially relevant factors to identify differences between target groups and tailor training content to their specific needs. The following study objectives were defined: 

The primary objectives of this study were:

1: To assess the level of knowledge among EMS personnel and emergency physicians regarding hand hygiene, basic hygiene measures, the use of PPE, and patient management following MDRO detection.
2: To identify key gaps in knowledge in order to inform the development of planned training formats.
3: To examine whether knowledge of basic hygiene measures, PPE use, and MDRO management is associated with WHO-based hand hygiene knowledge among EMS personnel and emergency physicians.
  The secondary objectives were:
4: To compare EMS personnel and emergency physicians with regard to:
  (a) WHO-based hand hygiene knowledge;
  (b) knowledge of basic hygiene measures, PPE use, and MDRO management;
  (c) digital health literacy;
  (d) teamwork and patient safety;
  (e) job satisfaction.
5: To assess the level of digital health literacy among EMS personnel and emergency physicians.
6: To identify sociodemographic and EMS-related factors associated with digital health literacy among these professionals.

## 2. Materials and Methods

### 2.1. Study Design and Period

A quantitative cross-sectional study was conducted. Data were collected from June to September 2025 using a standardised online questionnaire implemented in LimeSurvey (version 6.16.15+260330). As the survey was administered as an open web-based survey and distributed via a public link, participants were recruited through convenience sampling. Consequently, the sample cannot be considered representative of the overall EMS population, which limits the generalisability of the findings to EMS personnel and emergency physicians more broadly. 

The questionnaire included required fields. Participation was voluntary. No tokens or passwords were used. A pretest was conducted prior to data collection. The study received ethical approval, and a data availability statement was provided. Before starting the survey, participants were shown an informed consent statement. Participation was only possible after providing active consent by ticking a checkbox, and consent was recorded together with a timestamp. To preserve participant anonymity, no IP addresses or other personal identifiers were stored. Therefore, duplicate participation could not be technically prevented at the individual level. Instead, potential survey fraud and low-quality responses were assessed during data cleaning by checking for duplicate or highly similar entries, implausible response patterns, inconsistent eligibility information, and atypical completion times.

### 2.2. Study Setting, Participants, and Objectives

An a priori sample size calculation was performed for a two-sided independent-samples *t*-test comparing the WHO hand hygiene knowledge score between EMS personnel and emergency physicians. Assuming a medium effect size of d = 0.50, α = 0.05, a power of 0.80, and an allocation ratio of 1:1, 64 participants per group were required, resulting in a total required sample size of 128 participants. However, the planned sample size was not reached, and the final group sizes were unequal. Therefore, group comparisons should be interpreted with caution and regarded as exploratory.

The online questionnaire was used to assess knowledge of hygiene and infection prevention among EMS personnel and emergency physicians in Thuringia, Germany. Eligible participants were EMS personnel and emergency physicians affiliated with the six EMS agencies contacted and the local emergency physician network. In total, 353 potentially eligible individuals were invited to participate in the survey. Of these, 72 individuals responded to the questionnaire, corresponding to a response rate of 20.4%. 

Participants were invited via email distribution lists. EMS personnel were approached through the management of the respective EMS providers. The research team provided the management team with an invitation letter, the LimeSurvey link, and the informed consent form for distribution to eligible EMS personnel. Reminder letters were sent at 14-day intervals. Emergency physicians were invited through the lead emergency physician using the same procedure. The relatively low response rate may be partly explained by the indirect recruitment process via organisational gatekeepers, high workload, and shift-based work schedules among EMS personnel and emergency physicians, despite the use of reminder letters. After completion of data collection, the raw data were exported from LimeSurvey and prepared for statistical analysis using IBM SPSS Statistics, Version 29. During data preparation, variables were checked, coded, and recoded where necessary. Scale and sum scores for the measurement instruments used were calculated according to the respective scoring instructions.

Questionnaires were excluded if they lacked sufficient sociodemographic information and responses to the relevant measurement instruments, making meaningful classification or analysis impossible. Partially completed questionnaires were included in analyses whenever the required information for the respective analysis was available. Missing values were not imputed; therefore, sample sizes varied between individual analyses.

Because participation was voluntary, self-selection may have occurred, and participants may systematically differ from non-participants in their attitudes, prior knowledge, or motivation. Individuals without internet access may also be underrepresented. These factors limit the generalisability of the findings to the two target groups studied. Potential confounders, such as age, education, and prior knowledge, were considered; nevertheless, residual confounding and other unmeasured factors may still have influenced the results. The CHERRIES recommendations were used as a guide for reporting the online survey [10].

The primary aim of the study was to assess basic knowledge of hygiene and infection prevention among EMS personnel and emergency physicians. In addition, key contextual conditions and potentially relevant factors were examined, including digital health literacy, job satisfaction, teamwork, and patient safety.

### 2.3. Measures and Instruments

Hand hygiene knowledge was assessed using the WHO 2009 Hand Hygiene Knowledge Questionnaire for Health-Care Workers [11]. This instrument covers key aspects of hand hygiene, including the basic principles of pathogen transmission, situations in which hygienic hand disinfection should be performed, and knowledge of alcohol-based hand disinfection and handwashing [11]. The established German version of the questionnaire was used. Therefore, no additional forward-backward translation was conducted for this instrument. As the WHO instrument is a knowledge questionnaire covering different areas of hand hygiene knowledge and using different item formats, the interpretability of internal consistency is limited.

The WHO Hand Hygiene Knowledge Questionnaire for Health-Care Workers was scored by assigning one point for each correct answer and zero points for incorrect or missing answers. A total knowledge score was calculated. Based on percentage cut-off points commonly used in the literature, scores were categorised as poor (<50% correct), moderate (50–74% correct), and good (≥75% correct) [12]. 

In addition, an extended in-house hygiene knowledge test was used, covering practical content on basic hygiene measures, PPE, and the management of MDROs. As with the WHO questionnaire, knowledge in this test was assessed using a total score. The test comprised 37 items, with one point awarded for each correct answer; higher scores therefore reflected higher levels of knowledge (maximum 37 points). To facilitate interpretation, the total score was categorised into three groups based on percentage thresholds: poor (0–18 points, <50% correct), moderate (19–27 points, 50–74% correct), and good (28–37 points, ≥75% correct). These cut-offs were used as a pragmatic classification and are consistent with thresholds commonly applied in the literature, similar to Bloom’s cut-offs.

The extended hygiene knowledge test was developed in German by the team at the Institute for Infectious Diseases and Infection Control at University Hospital Jena and pilot-tested with physicians and infection control nurses. For the English manuscript, the German items and descriptions were translated into English by the research team; no formal back-translation was performed. Internal consistency was assessed for the dichotomous knowledge items, with Cronbach’s alpha = 0.61 for basic hygiene measures, 0.34 for PPE, and 0.63 for MDRO-related knowledge. The extended hygiene knowledge test was developed specifically for this study to assess baseline knowledge across selected hygiene-relevant domains prior to the planned training intervention. It was not designed as a formally validated psychometric instrument to measure a single underlying construct. Rather, it covered several content areas relevant to the EMS setting, including basic hygiene measures, PPE, and the management of patients with MDROs.

Digital health literacy was measured using the Digital Health Literacy Instrument (DHLI). Developed by van der Vaart and Drossaert (2017), the DHLI assesses “Health 1.0” and “Health 2.0” skills, such as information searching, contributing content, and evaluating information reliability [13]. The instrument was considered suitable for assessing the technical and critical appraisal skills relevant to Moodle-based training in the present study. As no established and universally validated DHLI thresholds are available, the categorisation system proposed by Dadaczynski et al. (2022) was adopted as a pragmatic approach to describe levels of digital health literacy [14]. Accordingly, the resulting categories should be interpreted as descriptive groupings rather than validated diagnostic cut-off values. Cronbach’s alpha values for the scales ranged from 0.65 to 0.75 [14]. Internal consistency was high in this study, with Cronbach’s alpha = 0.90.

Teamwork and patient safety were assessed using the Teamwork and Patient Safety Questionnaire (FTPS) [15]. Job satisfaction was assessed using the Short Questionnaire for Assessing General and Facet-Specific Job Satisfaction (KAFA) [16]. The FTPS is the German translation of the English-language Safety Attitudes Questionnaire (SAQ) [15]. Internal consistency, as measured by Cronbach’s alpha, ranged from 0.63 to 0.83 across the scales [15]. Internal consistency was high in this study, with Cronbach’s alpha = 0.90. To estimate average professional experience, the midpoint of each response category was used (e.g., 2.5 years for “<5 years” and 7 years for “5–9 years”). For the open-ended category “15 years or more,” 17.5 years was assumed.

### 2.4. Statistical Analysis

Results were described for the total sample and by occupational group (EMS personnel vs. emergency physicians) using frequencies, percentages, mean values, and total scores, as appropriate. Percentages were based on valid cases. Owing to item non-response, the number of valid cases varied across analyses; therefore, analyses were conducted using available-case data. For the WHO hand hygiene questionnaire and the in-house knowledge test, knowledge levels were described for the total sample and by occupational group using frequencies and percentages. Group differences in categorical variables were tested using Fisher’s exact test.

For item-level comparisons of correct responses between EMS personnel and emergency physicians, Fisher’s exact test was used because several 2 × 2 contingency tables included small or sparse cell counts. Given the conservative nature and limited statistical power of this test, the results were interpreted as exploratory and were not used as the sole basis for drawing conclusions.

For the DHLI, mean values and total scores were calculated for each dimension. Relationships between variables were analysed using Pearson and Spearman correlation analyses. Pearson’s correlation was used for metric variables, whereas Spearman’s correlation was applied to ordinal variables.

Independent-samples *t*-tests were used only for aggregated sum or mean scale scores to compare EMS personnel and emergency physicians. Approximate normality of these scale scores was assessed graphically prior to analysis.

A simple linear regression was conducted with the WHO hand hygiene knowledge sum score as the dependent variable and the sum score of the extended hygiene knowledge test on basic hygiene measures, PPE, and MDROs as the independent variable. Both variables were treated as metric scores. Prior to analysis, the assumptions of linear regression were assessed, including graphical checks of linearity, homoscedasticity, and approximate normality of residuals using residual and Q-Q plots.

To complement the bivariate group comparisons and adjust for selected potential confounders, an exploratory multivariable linear regression model was fitted with the WHO hand hygiene knowledge sum score as the dependent variable. Professional group was specified as the primary predictor, with professional experience and formal hand hygiene training within the preceding three years included as covariates. Professional group (0 = EMS personnel, 1 = emergency physicians) and prior formal hand hygiene training (0 = no, 1 = yes) were dummy-coded, whereas professional experience was entered as an ordinal variable. Given the limited sample size, the analysis was considered exploratory, and the resulting estimates were interpreted with appropriate caution.

Correlations between the DHLI total score and selected sociodemographic, internet-related, job satisfaction, teamwork, and patient safety variables were calculated using Pearson’s correlation coefficient for variables measured on an approximately metric scale assuming a linear relationship. For ordinal variables or variables not meeting these assumptions, Spearman’s rank correlation coefficient was used.

### 2.5. Limitations

All statistical analyses were conducted in an exploratory framework. As this survey used convenience sampling and was not designed to test a predefined confirmatory hypothesis family, no formal correction for multiple comparisons was applied. The reported *p*-values should therefore be understood as exploratory indicators of potential differences or associations rather than confirmatory evidence. Given the number of statistical tests performed, the possibility of type I error inflation was considered when interpreting the findings. 

Missing data should be considered when interpreting the findings. As missing values were not imputed and partially completed questionnaires were included only when the relevant data were available, bias may have occurred if missingness was not random. The substantial variation in valid responses across outcome measures indicates survey attrition and item- or instrument-level missingness. This was particularly pronounced for the job satisfaction scale, for which valid data were available for only 27 of 72 participants. Although respondent burden or survey fatigue related to questionnaire length and complexity may have contributed to this pattern, these factors were not formally assessed and therefore cannot be established as underlying causes. The resulting reduction in analysable observations, particularly for job satisfaction and subgroup analyses, reduced statistical power and increased the risk of type II errors. Moreover, the possibility of attrition-related selection bias cannot be excluded if participants who completed the later sections of the questionnaire differed systematically from those who did not. 

A further limitation concerns the difficulty and discriminatory power of individual knowledge items. Since some questions were answered correctly by all participants (e.g., individual items of the Hand Hygiene Knowledge Questionnaire), these items provided little information for distinguishing between participants with higher and lower levels of knowledge.

The categorisation of knowledge as poor, moderate, or good was based on percentage cut-offs commonly applied in previous studies rather than on thresholds formally established by the WHO. Accordingly, these categories should be regarded as pragmatic descriptive classifications rather than validated diagnostic cut-offs. Although the extended hygiene knowledge test was developed by infection prevention and control experts and pilot-tested prior to use, it did not undergo formal psychometric validation. Internal consistency was limited across the assessed content areas, particularly for PPE (Cronbach’s α = 0.34), and remained below the conventional threshold of 0.70 for basic hygiene (α = 0.61) and MDRO-related knowledge (α = 0.63). These findings suggest limited inter-item homogeneity and indicate that the items, particularly those addressing PPE, should not be interpreted as measuring a single underlying construct. This may, at least in part, reflect the intentionally heterogeneous and content-specific nature of the assessment. Nevertheless, the limited internal consistency and lack of formal validation constrain the interpretation of the total and content-area scores, as well as the derived knowledge categories. The findings should therefore be interpreted as descriptive and exploratory indicators of knowledge across selected hygiene-related domains rather than as validated measures of overall hygiene competence.

Self-assessed hygiene knowledge may be affected by cognitive bias, particularly social desirability and overestimation of one’s own knowledge, and therefore may not fully reflect actual knowledge levels.

Because several knowledge items were based on German infection prevention recommendations, such as KRINKO recommendations published by the Robert Koch Institute, their external validity may be limited in international healthcare systems with different regulatory frameworks. The generalisability of the findings is further limited by the single-state setting and the use of convenience sampling. EMS systems may vary substantially across federal states and countries in terms of organisational structures, professional roles, training requirements, infection prevention policies, and regulatory frameworks. Accordingly, the findings should be extrapolated to other EMS systems and international healthcare settings only with caution.

Although an exploratory multivariable regression model was fitted for WHO hand hygiene knowledge, the limited sample size restricted the number of potential confounders that could be included. Relevant contextual factors, including EMS agency-specific operational protocols, local training structures, and urban–rural differences within Thuringia, were not assessed and could therefore not be accounted for. Consequently, residual confounding cannot be excluded.

Some knowledge items may have addressed overlapping content, meaning that full item independence cannot be assumed. Because all items were equally weighted, potential inter-item correlations may have influenced the total knowledge score and its subsequent analyses.

Because several item-level comparisons were based on small and sparse 2 × 2 contingency tables, the statistical power to detect group differences was limited. Moreover, Fisher’s exact test is conservative; therefore, non-significant item-level results should not be interpreted as evidence of no difference between groups.

## 3. Results

### 3.1. Characteristics

The response rate was 20.4% (72 responses out of 353). Among the 72 participants in the online survey, 65.3% were male and 34.7% were female. A total of 72.2% of participants were in full-time employment, and just under 20% reported holding a management position. Participants comprised the following professional groups: emergency paramedics (43.1%), emergency medical technicians (6.9%), paramedics (22.2%), and emergency physicians (27.8%).

**Figure 1:**
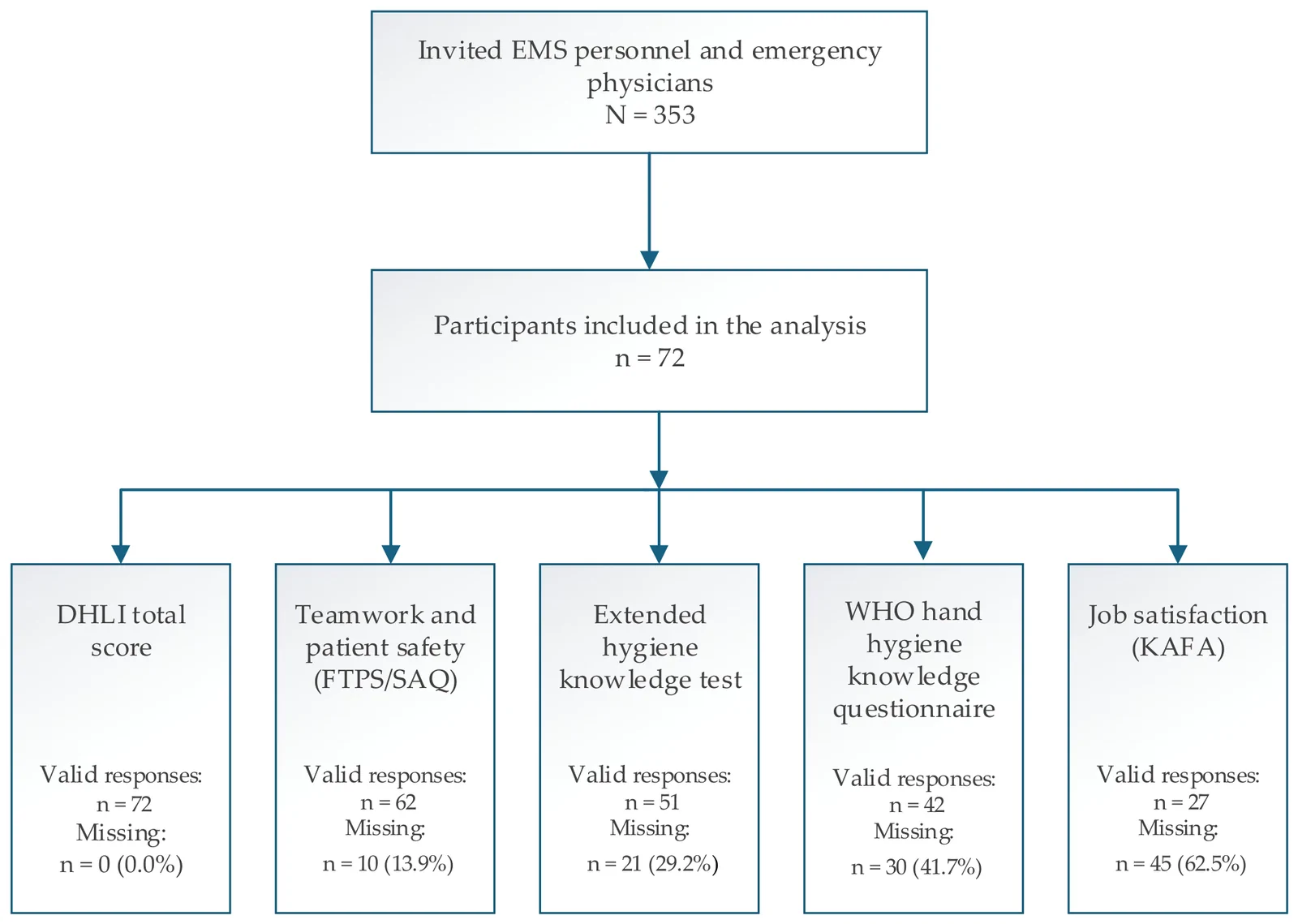
Participant flow and available-case samples across the main outcome measures.

The number of valid responses varied across the main outcome measures because partially completed questionnaires were retained whenever sufficient data were available for the respective analysis. Among the 72 included participants, valid data were available for the WHO hand hygiene knowledge questionnaire for 42 participants (58.3%), for the extended hygiene knowledge test covering basic hygiene measures, PPE, and MDRO management for 51 participants (70.8%), for the DHLI total score for 72 participants (100%), for job satisfaction for 27 participants (37.5%), and for teamwork and patient safety for 62 participants (86.1%). Missing data were not imputed, and all analyses were performed using the available data for the respective outcome. Participant flow and the number of valid responses for each main outcome measure are presented in Figure 1.

Accordingly, the denominators reported in the tables reflect the number of participants with valid data for the respective instrument or analysis and may therefore differ from the total included sample (n = 72).

More than half of the respondents (58.3%) worked in ambulances. The EMS personnel and emergency physicians were based in Thuringia, Germany. The mean age of the respondents was 36.99 years (SD = 12.63). A total of 31.9% reported 15 years or more of EMS experience, 26.4% reported 5–9 years, 16.7% reported 10–14 years, and 25.0% reported less than 5 years. The average professional experience across all respondents was 10.06 years (SD = 5.96). EMS personnel (n = 52) had a mean of 8.62 years (SD = 5.78) of professional experience, whereas emergency physicians (n = 20) reported an average of 13.80 years (SD = 4.77). The majority of respondents (97.2%) stated that their EMS provider had a hygiene plan including standard operating procedures for basic hygiene measures, PPE, and the management of patients with MDROs.

The majority of respondents (69.0%) stated that they had received formal training in hand disinfection within the last three years. Almost all of them (97.6%) reported routinely using alcohol-based disinfectants for hand disinfection. To support the development of online training on hygiene and infection prevention, respondents were also asked about their internet use, internet skills, and technical equipment. The majority of respondents (51.4%) used the internet daily for professional purposes, and a further 30.6% used it several times a week. Internet use several times a month was reported by 9.7% of the respondents, whereas 4.2% reported using it once a week or less. In a professional context, most respondents primarily accessed the internet via their smartphone (73.6%). Personal computers (PCs) (13.9%) and laptops (11.1%) were mentioned much less frequently, and tablets were used only rarely (1.4%). Most respondents rated their internet skills as good (58.3%), followed by very good (25.0%). A smaller proportion rated their skills as satisfactory (15.3%) or adequate (1.4%). Further details are presented in Table 1.

### 3.2. Status Quo of Hand Hygiene Knowledge

Information on self-assessed hand hygiene knowledge was available for 42 of the 72 participants (58.3%). Overall, the majority of respondents rated their knowledge as “good” (69.0%; 29/42). A further 21.4% (9/42) rated it as “satisfactory” and 9.5% (4/42) as “very good”. Group analysis showed that EMS personnel (n = 28) predominantly rated their hand hygiene knowledge as “good” (60.7%; 17/28), followed by “satisfactory” (25.0%; 7/28) and “very good” (14.3%; 4/28). Emergency physicians (n = 14) mostly rated their knowledge as “good” (85.7%; 12/14), while 14.3% (2/14) rated it as “satisfactory”. None of the emergency physicians selected the category “very good”.

Hand hygiene knowledge was assessed using the WHO questionnaire for healthcare workers and categorised according to the achieved score: poor: <50% (<13 points), moderate: 50–74% (13–18 points), and good: ≥75% (≥19 points) (Table 2). Evaluable data were available for 42 of 72 participants (58.3%). Overall, the majority of the respondents demonstrated a moderate level of knowledge about hand hygiene (25/42; 59.5%), while 38.1% (16/42) achieved a good level. Only 2.4% (1/42) had a poor level of knowledge about hand hygiene (Table 2). Among EMS personnel (n = 28), 67.9% (19/28) had moderate knowledge, 28.6% (8/28) had good knowledge, and 3.6% (1/28) had poor knowledge. Among emergency physicians (n = 14), 57.1% (8/14) achieved a good level, and 42.9% (6/14) achieved a moderate level, none had a poor level (Table 2).

The WHO hand hygiene questionnaire revealed significant group differences in two fundamental statements on pathogen transmission (Fisher’s exact test). Emergency physicians were more likely to agree that contaminated healthcare worker hands are the most important transmission route (100% vs. 50.0%; *p* = 0.001) and that pathogens present on or in the patient are the most common source of nosocomial infections (78.6% vs. 42.9%; *p* = 0.048).

With regard to indications for hand disinfection (e.g., before and after patient contact), both groups predominantly gave correct answers, and no further significant differences were observed. However, another group difference emerged in response to a question on the appropriate hand hygiene measure before palpating the abdomen: emergency physicians were more likely to select hand disinfection correctly (92.9% vs. 60.7%; *p* = 0.036). A borderline significant group difference was also observed for the statement that alcohol-based hand rub causes more skin dryness than handwashing (false) (*p* = 0.057).

There were no statistically significant differences between the groups (all *p* > 0.05) regarding statements comparing alcohol-based hand disinfection with hand washing (e.g., speed, skin dryness, effectiveness, and duration). Overall, the results suggest that both groups had moderate to good knowledge of hand hygiene, with emergency physicians demonstrating slightly better basic knowledge of pathogen transmission and correct hand hygiene measures prior to abdominal palpation (Table 3).

To complement the bivariate group comparisons, an exploratory multivariable linear regression model was fitted with the WHO hand hygiene knowledge sum score as the dependent variable (Table 4). Professional group, professional experience, and formal hand hygiene training within the preceding three years were included as predictors. The model included 42 participants with complete data and explained 18.4% of the variance in WHO hand hygiene knowledge scores, F(3, 38) = 2.85, *p* = 0.050, R^2^ = 0.184, adjusted R^2^ = 0.119 (Table 4). After adjustment for professional experience and prior formal hand hygiene training, professional group was significantly associated with WHO hand hygiene knowledge (b = 2.324, SE = 0.841, β = 0.448, *p* = 0.009, 95% CI [0.621, 4.027]), with emergency physicians scoring higher than EMS personnel. In contrast, neither professional experience (b = −0.464, SE = 0.322, β = −0.227, *p* = 0.157, 95% CI [−1.116, 0.187]) nor prior formal hand hygiene training (b = −1.274, SE = 0.807, β = −0.241, *p* = 0.123, 95% CI [−2.908, 0.360]) was significantly associated with WHO hand hygiene knowledge (Table 4). Variance inflation factors ranged from 1.087 to 1.227, indicating no problematic multicollinearity. Given the limited sample size, these findings should be interpreted as exploratory.

### 3.3. Status Quo Knowledge of Basic Hygiene Measures, Personal Protective Equipment, and Dealing with MDROs

For the extended hygiene knowledge test on basic hygiene measures, PPE use, and MDRO management, evaluable data were available for 51 of 72 participants (70.8%). Overall, 45.1% (23/51) had a moderate level of knowledge (19–27 points) and 43.1% (22/51) had a poor level of knowledge (0–18 points). Only 11.8% (6/51) achieved a good level of knowledge (28–37 points) (Table 5).

Among EMS personnel (n = 35), a low level of knowledge predominated (48.6%; 17/35), followed by moderate (42.9%; 15/35) and good (8.6%; 3/35) levels (Table 5). Among emergency physicians (n = 16), by contrast, a moderate level of knowledge predominated (50.0%; 8/16), whereas 31.3% (5/16) fell into the poor category and 18.8% (3/16) into the good category (Table 5).

The three content areas of the extended hygiene knowledge test, namely basic hygiene measures, PPE, and MDRO management, were analysed descriptively. Given the heterogeneous and content-specific nature of the assessment and the absence of formal psychometric validation, findings are presented as descriptive response patterns across selected knowledge domains rather than as validated subscale scores. In the extended hygiene knowledge test, the majority of responses were correct. In the domain of basic hygiene measures, questions on key routine aspects of wipe disinfection after each patient transport were very often answered correctly (e.g., handles/adjustment levers of the transport stretcher and RR cuff, 100% in each case). However, lower percentages of correct responses and some group differences were found for specific questions: the proportion of correct answers regarding the non-recommended spraying of large areas with alcohol pump spray was higher among EMS personnel than among emergency physicians (90.0% vs. 55.6%; *p* = 0.056). Overall, knowledge of waste disposal (in relation to LAGA 18) was low (17.2%).

Most questions on PPE components were answered correctly (e.g., Filtering Face Piece (FFP) masks 94.1%, safety goggles 91.2%). A statistically significant group difference was found for the statement that disposable medical gloves should be worn when contact with pathogen-containing material is possible: emergency physicians answered this correctly more often than EMS personnel (83.3% vs. 36.4%; *p* = 0.013). Overall, only 64.7% correctly stated the need for hand disinfection after removing disposable medical gloves. The proportion of correct responses was higher among emergency physicians than among EMS personnel (83.3% vs. 54.5%; *p* = 0.140).

In the area of infection transport and MDROs, some key statements were answered correctly by almost all participants (e.g., PPE use depending on disease/transmission route: 100% in both groups). At the same time, gaps in knowledge were evident in specific scenarios, such as transport involving 4-MRGN (multidrug-resistant Gram-negative bacteria) detected in wound swabs (22.2% correct). A pronounced group difference was observed for the statement that 3-MRGN or 4-MRGN detections must be treated with antibiotics (correct answer: No): EMS personnel answered this question correctly less often than emergency physicians (37.5% vs. 100%; *p* < 0.001).

A simple linear regression was conducted to examine the association between the extended hygiene knowledge test score on basic hygiene measures, PPE, and MDROs and WHO hand hygiene knowledge. The model was statistically significant, F(1, 35) = 5.52, *p* = 0.025, and explained 13.6% of the variance in WHO hand hygiene knowledge (R^2^ = 0.136; adjusted R^2^ = 0.112). The extended hygiene knowledge test score was positively associated with WHO hand hygiene knowledge (B = 0.150, SE = 0.064, β = 0.369, *t* = 2.35, *p* = 0.025, 95% CI [0.020, 0.279]). Accordingly, each additional point in the extended hygiene knowledge test was associated with an average increase of 0.15 points in the WHO hand hygiene knowledge score.

### 3.4. Level of Digital Health Literacy

The survey revealed high levels of digital health literacy in operational (98.6%; n = 71) and navigational (84.7%; n = 61) skills (Supplementary Materials Table S1). High levels were also frequently observed in the domains of contributing own content (80.6%; n = 58), searching for information (68.1%; n = 49), determining relevance (66.7%; n = 48) and protecting privacy (66.7%; n = 48). The lowest proportion of high scores was found in the dimension “assessing reliability” (44.4%; n = 32), which showed the highest proportion of low scores (15.3%; n = 11) (see Supplementary Materials Table S1).

EMS personnel predominantly scored highly in almost all dimensions, particularly operational skills (98.1%; n = 51) and navigational skills (92.3%; n = 48). Low scores were rare overall (0–9.6%). Emergency physicians (n = 20) showed high scores in operational skills (100%; n = 20), but lower proportions of high scores in several other dimensions: searching for information (55.0%; n = 11), protecting privacy (55.0%; n = 11), and assessing reliability (25.0%; n = 5), with 30.0% (n = 6) showing low scores for the latter domain (Supplementary Materials Table S1). Overall, compared with EMS personnel, emergency physicians more frequently showed medium or low levels of digital health literacy in selected subdomains.

Total scores and mean values were calculated for the DHLI dimensions (Table 6). The overall mean score for digital health literacy was M = 3.17 (SD = 0.39) (Table 6). The mean score for digital health literacy differed significantly between the two groups, t(70) = 4.04, *p* < 0.001, d = 1.06. The highest scores were found for operational (Total score: M = 11.17, SD = 1.25; M = 3.72, SD = 0.41) and navigational (Total score: M = 10.19, SD = 1.52; M = 3.40, SD = 0.50) skills (Table 6). Information searching (Total score: M = 9.24, SD = 1.65; M = 3.08, SD = 0.55), contributing own content (Total score: M = 9.54, SD = 1.74; M = 3.18, SD = 0.58), determining relevance (Total score: M = 8.96, SD = 1.62; M = 2.99, SD = 0.54), and protecting privacy (Total score: M = 9.04, SD = 1.92; M = 3.01, SD = 0.64) also yielded predominantly high scores. Scores for the reliability dimension were comparatively lower (Total score: M = 8.47, SD = 1.95; M = 2.82, SD = 0.65). Navigational skills (t(70) = 3.29, *p* = 0.002, d = 0.86), information searching (t(70) = 2.96, *p* = 0.004, d = 0.78), adding self-generated content (t(70) = 2.30, *p* = 0.024, d = 0.60), evaluating reliability (t(70) = 3.38, *p* = 0.001, d = 0.89), determining relevance (t(70) = 2.37, *p* = 0.020, d = 0.62), and protecting privacy (t(70) = 2.68, *p* = 0.009, d = 0.71) also differed significantly between EMS personnel and emergency physicians.

### 3.5. Job Satisfaction

Job satisfaction (KAFA) was generally high (overall satisfaction: M = 3.90; SD = 0.54; n = 27) (Table 7). When examined by occupational group, scores were predominantly high, with emergency physicians (M = 4.04; SD = 0.42) reporting slightly higher overall satisfaction than EMS personnel (M = 3.83; SD = 0.59). At the facet level, mean scores in both groups were particularly high for “Colleagues” (EMS personnel: M = 4.01; SD = 0.67; emergency physicians: M = 4.13; SD = 0.24) and “Activities” (3.89; SD = 0.66 vs. 4.27; SD = 0.51) (Table 7). The facet “Development opportunities” had the lowest scores and fell in the mid-range for EMS personnel (M = 3.27; SD = 1.13), whereas emergency physicians reported higher scores (M = 3.91; SD = 0.56) (Table 7). The “Pay” facet also showed predominantly high scores (3.86; SD = 0.81 vs. 4.09; SD = 0.43). For the “Supervisors” facet, EMS personnel reported higher scores (M = 4.13; SD = 0.43) than emergency physicians (M = 3.78; SD = 0.94) (Table 7).

### 3.6. Teamwork and Patient Safety

Overall, 62 participants provided predominantly positive assessments of teamwork and patient safety on the FTPS/SAQ. The communication subscale received the highest scores (M = 3.95; SD = 0.48), followed by leadership (M = 3.68; SD = 0.61) and cooperation (M = 3.64; SD = 0.48) (Table 8). Handling errors also received positive ratings (M = 3.53; SD = 0.57). Group analysis revealed that EMS personnel and emergency physicians had similar scores for handling errors (M = 3.52 vs. M = 3.56), leadership (M = 3.68 vs. M = 3.69), and cooperation (M = 3.63 vs. M = 3.68) (Table 8). The only statistically significant difference (t(61) = 2.91, *p* = 0.005, d = 0.81) was found for communication, which was rated higher by EMS personnel (M = 4.06; SD = 0.46) than by emergency physicians (M = 3.69; SD = 0.45) (Table 8). Overall, the results indicated a supportive team and safety culture, although communication was rated less positively by emergency physicians.

### 3.7. Correlations Between Digital Health Literacy and Sociodemographic Factors, Job Satisfaction, Teamwork and Patient Safety

As expected, the correlation analysis revealed moderate to high positive correlations between the DHLI subscales and the total DHLI score (r = 0.511–0.789, *p* < 0.001 for all; n = 72). Table 9 shows predominantly weak to moderate correlations between digital health literacy (DHLI total score) and selected variables. Exploratory correlation analyses showed negative associations between DHLI scores and age (r = −0.255, *p* = 0.030) as well as professional experience (r = −0.247, *p* = 0.036) (Table 9). These findings suggest that digital health literacy tended to be lower among older and more experienced participants. However, due to the exploratory design and the limited sample size, these results should be interpreted cautiously.

Conversely, there was a moderate positive correlation between DHLI and self-assessed internet skills (r = 0.435; *p* < 0.001) (Table 9). No statistically significant correlations were found for internet use (r = 0.065, *p* = 0.065) or technical equipment (r = 0.086, *p* = 0.470). Regarding job satisfaction (KAFA), only pay was positively and statistically significantly associated with DHLI (r = 0.383; *p* = 0.048) (Table 9).

Because valid data for job satisfaction were available for only 27 participants, correlations involving the KAFA should be considered exploratory and interpreted with particular caution due to the limited sample size and resulting uncertainty of the estimates. These analyses were conducted to identify potential patterns of association and should not be interpreted as providing robust or generalisable evidence.

In addition, the DHLI subscale “Contributing own content” showed positive correlations with the KAFA dimensions “Development opportunities” (r = 0.443; *p* = 0.021) and “Pay” (r = 0.399; *p* = 0.039), as well as with overall satisfaction (r = 0.478; *p* = 0.012). Furthermore, the DHLI subscale “Determining relevance” correlated positively with the KAFA dimension “Supervisor” (r = 0.473; *p* = 0.013). All other correlations between DHLI and KAFA facets were not statistically significant (*p* > 0.05). The communication subscale showed a statistically significant positive correlation with teamwork and patient safety (FTPS) (r = 0.351; *p* = 0.005), whereas the other FTPS subscales did not show statistically significant correlations.

## 4. Implementation Plan Based on Study Findings

This study assessed hygiene knowledge and digital health literacy among EMS personnel and emergency physicians as a baseline assessment. Based on the findings, two training formats on hygiene and infection prevention will be developed for the two participant groups: face-to-face training for EMS personnel and online training for EMS personnel and emergency physicians. Because of the limited availability of emergency physicians, training for this survey group will be provided exclusively in an online format. First, an interdisciplinary team will develop a structured face-to-face seminar based on a train-the-trainer approach [17]. Selected trainers will be qualified to deliver hygiene and infection prevention content to EMS personnel in a standardised manner and subsequently disseminate this knowledge within their teams. Second, EMS personnel and emergency physicians will be provided with online training via the Moodle learning platform. This approach is intended to promote self-directed learning and to allow flexible participation regardless of time and location.

The review by Fernandes et al. [18] showed that digital learning formats were particularly effective in conveying hand hygiene knowledge, especially when incorporating visual elements (e.g., videos and animations), interactive elements (e.g., quizzes and gamification), and feedback-based components (e.g., providing immediate feedback) [18]. Several of the included studies explicitly reported improvements in theoretical knowledge following digital interventions, such as video-based training [18]. Against this background, the present study also examined respondents’ digital health, as this may influence the use and effectiveness of online learning opportunities.

Together, these two training formats are intended to disseminate knowledge rapidly and comprehensively, provide long-term support to emergency services, and strengthen resilience in future pandemic and crisis situations. A 2024 needs assessment among the heads of public health authorities in Germany showed that training courses and outreach teams could support and relieve services during future crises and pandemics [19].

## 5. Discussion

### 5.1. Level of Knowledge About Hand Hygiene, Basic Hygiene Measures, PPE, and MDROs

The findings on WHO hand hygiene knowledge indicated an overall moderate to good level of knowledge. Only a small number of respondents showed a poor level of knowledge, whereas a considerable proportion achieved a good level. At the same time, there were significant content-related differences between the survey groups: emergency physicians answered key statements on pathogen transmission correctly more frequently than EMS personnel. Emergency physicians also demonstrated greater knowledge regarding the correct hand hygiene measures before abdominal palpation. In contrast, both groups showed very high proportions of correct answers for classic indications for hand disinfection (e.g., before/after patient contact), suggesting that the issue was less a general lack of knowledge than uncertainty regarding specific core concepts.

In the extended hygiene knowledge test on basic hygiene measures, PPE, and MDRO management, by contrast, a weak to moderate level of knowledge was more common. A good level of knowledge was rarely achieved. Although questions addressing routine hygiene practices, such as wipe disinfection of key contact surfaces after patient transport, were generally answered correctly, the observed response patterns suggested potential knowledge gaps in more specific areas. These included regulatory and procedural aspects (e.g., waste disposal/LAGA 18 reference), the indication for the use of disposable medical gloves, and MDRO-related scenarios. Particularly relevant in practice was the misconception that 3- and 4-MRGN findings (MDRO) must always be “treated” with antibiotics, with emergency physicians responding correctly significantly more often. Likewise, the proportion of correct responses was low for specific transport scenarios (e.g., 4-MRGN detection in wound swabs). Overall, these patterns suggest that the main learning needs concern not the most basic actions themselves, but rather regulatory knowledge, confidence in indication-based decisions, and the ability to act appropriately in specific operational situations.

However, the findings from the extended hygiene knowledge test should be interpreted with caution. The instrument was developed specifically for this study to assess selected aspects of basic hygiene, PPE use, and the management of patients with MDROs in the EMS setting and did not undergo formal psychometric validation. Accordingly, it should be regarded as a study-specific assessment of selected factual hygiene knowledge rather than as a validated measure of overall hygiene competence.

This limitation is particularly relevant to the PPE-related findings, for which internal consistency was low. Given the limited inter-item homogeneity, PPE-related results should not be interpreted as representing a reliable subscale or a single underlying knowledge construct. Instead, individual responses should be considered descriptive, item-level findings that may indicate uncertainty in specific areas, such as indications for glove use or the need for hand disinfection following glove removal. Similar caution is warranted when interpreting the basic hygiene and MDRO content areas. Although their internal consistency was higher than that observed for PPE, it remained below the conventional threshold of 0.70. 

Despite a predominantly positive self-assessment of hand hygiene knowledge, previous studies have repeatedly shown knowledge gaps in key aspects of pathogen transmission and the basic principles of infection prevention [20]. At the same time, structured, competency-based training in EMS has been described as inconsistent or, in some cases, lacking [20]. Our study confirmed this pattern: although WHO-based hand hygiene knowledge was generally moderate to good, uncertainties and group differences became apparent with regard to basic principles of pathogen transmission and specific indications. These gaps were even more evident in the broader knowledge test on basic hygiene measures, PPE, and MDROs, in which the majority of respondents achieved only low to moderate scores and, in particular, MDRO-related scenarios and PPE indications were not consistently answered correctly. 

The exploratory multivariable regression analysis suggested that the observed difference in WHO hand hygiene knowledge between EMS personnel and emergency physicians persisted after adjustment for professional experience and prior formal hand hygiene training. However, given the limited number of complete cases and the small and unequal group sizes, these findings should be interpreted with caution. The absence of statistically significant associations for professional experience and prior training should not be interpreted as evidence of no effect, particularly given the limited statistical power. Accordingly, the observed association between professional group and WHO hand hygiene knowledge should be regarded as hypothesis-generating rather than as definitive evidence of systematically higher hygiene knowledge among emergency physicians.

In line with this, observational studies in EMS reported very low levels of actual hand hygiene compliance [20]. An Australian study showed that hand hygiene was performed in only approximately 6% of cases [20]. In addition, problematic overuse and continuous wearing of disposable medical gloves without changing them were observed, even when staff were in contact with equipment and environmental surfaces [20]. Other studies reported that only 48% of EMS personnel donned new gloves before patient contact and that 64% used the same gloves for multiple indications, which is particularly critical before aseptic procedures [4]. Taken together, these findings may indicate that high self-assessed knowledge does not necessarily correspond to reliable knowledge or guideline-adherent practice. Accordingly, future training formats could place greater emphasis on safe indication-based decision-making, appropriate glove use, and the principle of pathogen transmission in prehospital workflows.

Against this background, the findings may inform the development of targeted educational interventions, potentially including a combination of face-to-face train-the-trainer sessions and online training via Moodle. Case-based elements, such as short vignettes addressing infection transport, PPE selection, MDRO management, and disposal procedures, could be considered to address the identified areas of uncertainty more specifically. However, the present study primarily assessed the knowledge dimension of hygiene and infection prevention. Knowledge does not necessarily translate into practical skills, clinical problem solving, attitudes, or actual behaviour. Therefore, the findings should be interpreted as indicators of knowledge rather than evidence of practical competence or guideline adherence in real-world EMS settings. Future studies should complement knowledge tests with additional assessment formats, such as simulation-based testing, skills assessments, or direct observation of hygiene behaviour.

One limitation that should be acknowledged is that sample sizes varied across scales and that the findings were based on self-reported data collected in an online format.

Furthermore, the relatively low response rate, smaller-than-planned sample size, and unequal group sizes limited statistical power and representativeness. Although the a priori sample size calculation indicated that 128 participants (64 per group) were required, the final sample included 72 participants (52 EMS personnel and 20 emergency physicians). This reduced and imbalanced sample limited the strength of comparative analyses, including *t*-tests and Fisher’s exact tests, and increased the risk of type II errors. Therefore, non-significant findings should not be interpreted as evidence of no difference between groups. Because numerous statistical comparisons were performed without formal adjustment for multiple testing, significant *p*-values should be regarded as exploratory indicators rather than confirmatory evidence and require confirmation in larger, adequately powered studies. The findings may provide useful pilot data for the development of hygiene and infection prevention training in the EMS setting. However, their transferability to other regions and EMS systems should be considered limited. Since the study was conducted in one German federal state and included a convenience sample, the observed knowledge patterns may partly reflect local training structures, organisational routines, and German infection prevention recommendations. Thus, the results should be understood as context-specific preliminary findings that require confirmation in larger and more diverse EMS populations.

### 5.2. Digital Health Literacy

The observed correlation patterns supported the internal structure of the DHLI, as the subscales were consistently associated with the DHLI total score. This indicates that the scales reflected a common overarching construct of digital health literacy while also capturing distinct subdomains of digital health literacy.

The results on digital health literacy indicated an overall high level of competence in the EMS context, particularly with regard to basic application skills. Very high scores were observed for operational and navigational skills, suggesting that digital applications can generally be used confidently in everyday work and that information can usually be located efficiently on digital platforms. At the same time, the dimension “assessing reliability” showed comparatively lower scores and had the highest proportion of low scores. This may suggest that critical appraisal of information, rather than merely accessing digital resources, could be an area for further development. This is highly relevant for Moodle-based training, since learning in online environments depends strongly on the ability to evaluate sources, identify evidence-based information, and avoid misinformation.

Previous studies have shown a strong positive correlation between higher digital health literacy and a better understanding of e-learning and online training formats, while well-designed online training has also been shown to improve digital health literacy and the understanding of health information [21,22,23,24].

When the occupational groups were considered separately, EMS personnel scored predominantly high across almost all DHLI dimensions. Emergency physicians, despite uniformly high operational skills, more frequently showed medium or low scores in several DHLI dimensions, particularly for assessing reliability and, in some cases, information searching and privacy protection. These differences suggest that training formats should be adapted to the needs of the respective target groups. In the context of the planned Moodle training, this could be addressed by giving greater emphasis to critical source appraisal, classification of digital information, and data protection issues, linked to short, case-based learning tasks.

Within the face-to-face train-the-trainer approach, participants should therefore be taught not only hygiene-related content but also how to use reliable, quality-assured digital sources (e.g., guidelines, SOPs, professional associations) and how to act as peer trainers who disseminate consistent, reliable information. For Moodle, short, modular learning units with clear key messages, linked primary sources, checklists, and case vignettes appear appropriate. In addition, formative knowledge checks with feedback would be useful for systematically developing critical appraisal and decision-making skills.

The correlation analyses complemented this picture: higher DHLI scores were associated with better self-assessed internet skills and were negatively correlated with age and professional experience. This suggests that digital competencies may vary by age, professional experience, educational background, and professional role. Emergency physicians and EMS personnel differ in training pathways, responsibilities, and routine use of digital medical information sources, which may influence how digital information is searched for, evaluated, and applied in practice. Accordingly, online training should be accessible, time-efficient, and suitable for heterogeneous skill levels, for example through clear navigation, short learning units, mobile usability, and repeatable microlearning activities.

### 5.3. Relationship Between Digital Health Literacy and Job Satisfaction

The job satisfaction data revealed positive correlations between digital health literacy and selected aspects of job satisfaction. Higher levels of digital health literacy were associated with higher satisfaction scores in specific areas, particularly remuneration and development opportunities. In addition, the DHLI dimension of “determining relevance” correlated positively with satisfaction with one’s supervisor. These findings suggest that more advanced digital skills, especially creating/sharing content and evaluating the relevance of digital information, may be associated with more favourable perceptions of work-related conditions.

Given the very small number of valid cases for job satisfaction (n = 27), the exploratory design, and the number of statistical comparisons performed, associations between digital health literacy and job satisfaction should be interpreted as unstable, hypothesis-generating patterns that require confirmation in larger samples with more complete data. Similarly, the observed positive association between DHLI and the communication subscale of the FTPS should be viewed as exploratory rather than as robust evidence of a relationship between digital information and communication skills and team-related processes.

### 5.4. Job Satisfaction, Teamwork, and Patient Safety

The results on job satisfaction showed an overall high level of satisfaction in the sample, both among EMS personnel and emergency physicians. In both groups, the facets “colleagues” and “activities” received particularly positive ratings, indicating stable social resources and a general perception of work in EMS as meaningful. At the same time, specific areas for development also emerged: “development opportunities” received only mid-range scores, particularly among EMS personnel, suggesting a potential organisational starting point that goes beyond the mere transfer of knowledge.

Group-specific differences were also evident in ratings of supervisors, with EMS personnel reporting higher ratings than emergency physicians. This may reflect differences in experiences with leadership structures and involvement in decision-making processes. Overall, the high job satisfaction scores are consistent with the assumption that a generally positive work environment may facilitate willingness to learn and the perceived applicability of training content. However, structural factors, particularly development opportunities, should also be taken into account as contextual conditions.

The findings on teamwork and patient safety (SAQ/FTPS) indicated a predominantly supportive team and safety culture. The highest scores were observed for communication, followed by leadership and cooperation, while handling errors was also rated positively. EMS personnel and emergency physicians showed similar ratings for error management, leadership, and cooperation. The observed difference in communication, although statistically significant and accompanied by a large effect size, should be interpreted cautiously because of the exploratory design, multiple testing, small sample size, and low response rate. This finding may indicate that emergency physicians perceived communication processes in the prehospital setting, such as handovers, interprofessional coordination, and role clarity, as less consistently supportive, and communication-related aspects could therefore be considered in future training formats.

A systematic review showed that teamwork and communication training, including TeamSTEPPS and crew resource management (CRM), often simulation-based and accompanied by structured debriefing, repeatedly improved important dimensions of safety culture such as communication, team climate, and handovers [25].

Because the present study assessed knowledge and perceptions rather than the effectiveness of specific educational formats, approaches such as train-the-trainer sessions or Moodle-based training should be evaluated in future intervention studies, including strategies to reinforce learning over time.

## 6. Conclusions

This exploratory pilot study provides preliminary indications of potential knowledge gaps in hygiene and infection prevention among EMS professionals. While WHO-based hand hygiene knowledge was generally moderate to good, the broader assessment of basic hygiene measures, PPE use, and MDRO management identified specific areas of uncertainty that may represent targets for future educational interventions. Given the study-specific and unvalidated nature of the extended hygiene knowledge assessment, its limited internal consistency, and the methodological constraints of the study, these findings should be considered exploratory and hypothesis-generating rather than definitive evidence of overall hygiene competence or occupational group differences. 

Future studies should confirm these findings in larger, adequately powered, and more representative multicentre samples using validated or further psychometrically evaluated assessment instruments. Longitudinal intervention studies incorporating pre–post assessments and longer-term follow-up are warranted to determine whether targeted educational strategies can produce sustained improvements in hygiene-related knowledge and practice in EMS settings. Such evidence may ultimately support the development of effective, context-specific infection prevention strategies and contribute to improving the quality and safety of prehospital patient care.

## Figures and Tables

**Table 1:** Characteristics of the study population (n = 72).

Variable	Sample (n = 72)
Age, M (SD)	36.99 (12.63)
Sex	
Female, n (%)	25 (34.7)
Male, n (%)	47 (65.3)
Professional group	
EMS personnel, n (%)	52 (72.2)
Emergency physicians, n (%)	20 (27.8)
Years of professional experience	
<5 years, n (%)	18 (25.0)
5–9 years, n (%)	19 (26.4)
10–14 years, n (%)	12 (16.7)
≥15 years, n (%)	23 (31.9)
Leadership role	
Yes, n (%)	14 (19.4)
No, n (%)	58 (80.6)
Employment status	
Full-time, n (%)	52 (72.2)
Part-time, n (%)	14 (19.4)
Other, n (%)	6 (8.4)
Primary vehicle type	
Ambulance, n (%)	42 (58.3)
Emergency physician response vehicle, n (%)	21 (29.2)
Patient transport ambulance, n (%)	5 (6.9)
Intensive care transport ambulance, n (%)	1 (1.4)
Other, n (%)	3 (4.2)
Hygiene plan available	
Yes, n (%)	70 (97.2)
No, n (%)	2 (2.8)
Formal hand hygiene training in the past 3 years	
Yes, n (%)	29 (69.0)
No, n (%)	13 (31.0)
Self-rated hand hygiene knowledge	
Very good, n (%)	4 (9.5)
Good, n (%)	29 (69.0)
Satisfactory, n (%)	9 (21.4)
Internet use for professional purposes	
Less often, n (%)	3 (4.2)
Several times per month, n (%)	7 (9.7)
Once per week, n (%)	3 (4.2)
Several times per week, n (%)	22 (30.6)
Daily, n (%)	37 (51.4)
Self-rated internet skills	
Very good, n (%)	18 (25.0)
Good, n (%)	42 (58.3)
Satisfactory, n (%)	11 (15.3)
Adequate, n (%)	1 (1.4)
Technical equipment in EMS setting	
Poor: access to modern digital devices/software is very limited, n (%)	5 (6.9)
Adequate: access to basic digital devices/software, n (%)	24 (33.3)
Good: access to modern devices/software, n (%)	35 (48.6)
Very good: access to the latest devices/software, n (%)	8 (11.1)

Note: Valid n refers to participants with complete data for the respective instrument.

**Table 2:** Hand hygiene knowledge level assessed using the WHO Hand Hygiene Knowledge Questionnaire for Health-Care Workers (n = 42).

Hand Hygiene Knowledge Level (Score Range)	EMS Personnel (n = 28)	Emergency Physicians (n = 14)	Total (n = 42)
Low: <50% (<13 points)	1 (3.6%)	0 (0%)	1 (2.4%)
Moderate: 50–74% (13–18 points)	19 (67.9%)	6 (42.9%)	25 (59.5%)
Good: ≥75% (≥19 points)	8 (28.6%)	8 (57.1%)	16 (38.1%)

Note: Valid n refers to participants with complete data for this instrument.

**Table 3:** WHO hand hygiene knowledge among EMS personnel and emergency physicians in healthcare settings (n = 42).

Item	EMS Personnel n/N (%)	Emergency Physicians n/N (%)	Total n/N (%)	*p*-Value
The main route of transmission of potentially dangerous pathogens between patients in healthcare facilities is through the hands of healthcare workers contaminated with pathogens.	14/28 (50.0)	14/14 (100)	28/42 (66.7)	0.001
Pathogenic germs already present on or within the patient is the most frequent source of pathogenic germs responsible for healthcare associated infections (e.g., in the hospital)	12/28 (42.9)	11/14 (78.6)	23/42 (54.8)	0.048
**The following hand hygiene measures prevent the transmission of pathogens to patients; answer with yes or no**				
Hygienic hand disinfection before patient contact (*Yes*)	28/28 (100)	14/14 (100)	42/42 (100)	N/A
Hygienic hand disinfection immediately after risk of exposure to body fluids (*No*)	4/28 (14.3)	2/14 (14.3)	6/42 (14.3)	1.00
Hygienic hand disinfection after exposure to the patient’s immediate surroundings (*No*)	7/28 (25.0)	4/14 (28.6)	11/42 (26.2)	1.00
Hygienic hand disinfection immediately before an aseptic procedure (*Yes*)	28/28 (100)	13/14 (92.9)	41/42 (97.6)	0.333
**The following hand hygiene actions prevent the transmission of pathogenic germs to the healthcare worker, answered with yes or no**				
Hygienic hand disinfection after touching a patient (*Yes*)	28/28 (100)	14/14 (100)	42/42 (100)	N/A
Hygienic hand disinfection immediately after risk of exposure to body fluids (*Yes*)	27/28 (96.4)	14/14 (100)	41/42 (97.6)	1.00
Hygienic hand disinfection immediately before an aseptic procedure (*No*)	8/28 (28.6)	2/14 (14.3)	10/42 (23.8)	0.451
Hygienic hand disinfection after exposure to the patient’s immediate surroundings (*Yes*)	25/28 (89.3)	14/14 (100)	39/42 (92.9)	0.539
**True statements about alcohol-based disinfectant and hand washing with soap and water are answered with true or false**				
Hand disinfectant is more rapid for hand cleansing than hand washing (*True*)	17/28 (60.7)	9/14 (64.3)	26/42 (61.9)	1.00
Hand disinfectant causes skin dryness more than hand washing (*False*)	13/28 (46.4)	11/14 (78.6)	24/42 (57.1)	0.057
Hand disinfectant is more effective against pathogenic germs than hand washing (*True*)	21/28 (75.0)	14/14 (100)	35/42 (83.3)	0.075
Hand washing and hand disinfectant are recommended to be performed in sequence (*False*)	14/14 (50.0)	5/14 (35.7)	19/42 (45.2)	0.515
Duration of hygienic hand disinfection (*30 s.*)	18/28 (64.3)	10/14 (71.4)	28/42 (66.7)	0.738
**Type of hand hygiene method required, answered with the alternatives: hand disinfection, hand washing, or none**				
Before palpation of the (*hand disinfection*)	17/28 (60.7)	13/14 (92.9)	30/42 (71.4)	0.036
Before giving an injection (*hand disinfection*)	28/28 (100)	14/14 (100)	42/42 (100)	N/A
After emptying a bedpan (*hand washing*)	7/28 (25.0)	4/14 (28.6)	11/42 (26.2)	1.00
After removing disposable medical gloves (*hand disinfection*)	21/28 (75.0)	9/14 (64.3)	30/42 (71.4)	0.491
After making a patient’s bed (*hand disinfection*)	23/28 (82.1)	12/14 (85.7)	35/42 (83.3)	1.00
After visible contact with blood (*hand washing*)	11/28 (39.3)	6/14 (42.9)	17/42 (40.5)	1.00
**The following aspects should be avoided to prevent** **the colonization of hands with pathogenic germs; answered with yes or no**				
Wearing jewellery (*Yes*)	28/28 (100)	14/14 (100)	42/42 (100)	N/A
Damaged skin (*Yes*)	28/28 (100)	14/14 (100)	42/42 (100)	N/A
Artificial fingernails (*Yes*)	28/28 (100)	14/14 (100)	42/42 (100)	N/A
Regular use of hand cream (*No*)	26/28 (92.9)	12/14 (85.7)	38/42 (90.5)	0.590

Note: Correct answers are shown in italics. Data are presented as the number of correct answers (n) relative to the total number of respondents (N), in the format n/N (%); n = 42. Valid n refers to participants with complete data for this instrument.

**Table 4:** Exploratory multivariable linear regression model with WHO hand hygiene knowledge score as the dependent variable (n = 42).

Dependent Variable: WHO Hand Hygiene Knowledge Score							
Coefficients	b	SE	β	t	*p*	95%-CI	
						LL	UL
(Constant)	19.046	1.050	-	18.146	<0.001	16.922	21.171
Professional group	2.324	0.841	0.448	2.762	0.009	0.621	4.027
Professional experience	−0.464	0.322	−0.227	−1.442	0.157	−1.116	0.187
Prior formal hand hygiene training	−1.274	0.807	−0.241	−1.578	0.123	−2.908	0.360

Note: n = 42; R^2^ = 0.184; adjusted R^2^ = 0.119; F(3, 38) = 2.85; *p* = 0.050. Valid n refers to participants with complete data for this instrument.

**Table 5:** Hygiene knowledge levels for basic hygiene measures, PPE use, and MDRO management by occupational group (n = 51).

Hygiene Knowledge Level (Score Range)	EMS Personnel (n = 35), n (%)	Emergency Physicians (n = 16), n (%)	Total (n = 51), n (%)
Low (0–18 points; <50%)	17 (48.6)	5 (31.3)	22 (43.1)
Moderate (19–27 points; 50–74%)	15 (42.9)	8 (50.0)	23 (45.1)
Good (28–37 points; ≥75%)	3 (8.6)	3 (18.8)	6 (11.8)

Note: Valid n refers to participants with complete data for this instrument.

**Table 6:** Total score and mean scores of the DHLI dimensions (n = 72).

Digital Health Literacy Skill (n = 72)	Total Score		Mean	
	M	SD	M	SD
Navigational skills	10.19	1.52	3.40	0.50
Operational skills	11.17	1.25	3.72	0.41
Information searching	9.24	1.65	3.08	0.55
Adding self-generated content	9.54	1.74	3.18	0.58
Evaluating reliability	8.47	1.95	2.82	0.65
Determining relevance	8.96	1.62	2.99	0.54
Protecting privacy	9.04	1.92	3.01	0.64
Total digital health literacy	-	-	3.17	0.39

Note: Items were rated on a 4-point scale (“very easy”–“very difficult”). Scores were reverse-coded so that higher values indicate greater digital health literacy. Total score: high DHL: 9–12, medium DHL: 7–8, low DHL: 3–6. Valid n refers to participants with complete data for this instrument.

**Table 7:** Short Questionnaire for Assessing General and Facet-Specific Job Satisfaction (KAFA) (n = 27).

KAFA-Scale	EMS Personnel (n = 18)		Emergency Physicians (n = 9)		Total (n = 27)	
Facets	M	SD	M	SD	M	SD
Activities	3.89	0.66	4.27	0.51	4.01	0.63
Colleagues	4.01	0.67	4.13	0.24	4.05	0.56
Development opportunities	3.27	1.13	3.91	0.56	3.48	1.01
Pay	3.86	0.81	4.09	0.43	3.93	0.71
Supervisor	4.13	0.43	3.78	0.94	4.01	0.65
Overall satisfaction	3.83	0.59	4.04	0.42	3.90	0.54

Note: Response scale: 1–2 = low job satisfaction; 3 = moderate/neutral; 4–5 = high job satisfaction. Valid n refers to participants with complete data for this instrument.

**Table 8:** FTPS/SAQ teamwork and patient safety scores: group comparisons between EMS personnel and emergency physicians (n = 62).

FTPS/SAQ-Subscale	EMS Personnel M (SD)	Emergency Physicians M (SD)	Total M (SD)	*p*	d
Dealing with mistakes	3.52 (0.62)	3.56 (0.43)	3.53 (0.57)	0.796	-
Leadership	3.68 (0.63)	3.69 (0.58)	3.68 (0.61)	0.936	-
Cooperation	3.63 (0.53)	3.68 (0.37)	3.64 (0.49)	0.757	-
Communication	4.06 (0.46)	3.69 (0.45)	3.95 (0.48)	0.005	0.81

Note: Scale 1 “definitely does not apply” to 5 “definitely applies”. Valid n refers to participants with complete data for this instrument.

**Table 9:** Correlations of digital health literacy with sociodemographics, internet use, job satisfaction, teamwork, and patient safety.

Variable Assessed	Correlation Coefficient r/ρ	*p*
Age	−0.255 ^b^	0.030
Internet usage	0.065 ^a^	0.065
Internet skills	0.435 ^a^	<0.001
Technical equipment	0.086 ^a^	0.470
Professional experience	−0.247 ^a^	0.036
KAFA dimension Activities	0.118 ^b^	0.558
KAFA dimension Work colleagues	0.082 ^b^	0.683
KAFA dimension Development opportunities	0.185 ^b^	0.356
KAFA dimension Pay	0.383 ^b^	0.048
KAFA dimension Supervisor	0.172 ^b^	0.390
KAFA overall satisfaction	0.254 ^b^	0.202
FTPS subscale Dealing with mistakes	0.020 ^b^	0.875
FTPS subscale Management	0.192 ^b^	0.135
FTPS subscale Cooperation	0.203 ^b^	0.110
FTPS subscale Communication	0.351 ^b^	0.005

Note: Spearman rho ^a^, Pearson ^b^. Valid n varied by instrument: sociodemographic and internet-related variables, n = 72; KAFA, n = 27; and FTPS/SAQ, n = 62.

## Data Availability

The data used and/or analysed during the current study are available from the corresponding author on reasonable request. The data is not publicly accessible due to data protection and privacy-related restrictions.

## Supplementary Materials

The following supporting information can be downloaded at: https://www.mdpi.com/article/10.3390/healthcare14183091/s1, Table S1: DHLI dimensions by occupational group: proportions of high, medium, and low digital health literacy (n = 72).

## Abbreviations

ABHR	Alcohol-based hand rub
CI	Confidence interval
DHLI	Digital Health Literacy Instrument
DHL	Digital health literacy
ECDC	European Centre for Disease Prevention and Control
EEA	European Economic Area
EMS	Emergency medical services
ESBL	Extended-spectrum β-lactamase
FFP	Filtering facepiece respirator
FTPS	Teamwork and Patient Safety Questionnaire
HAI	Health care-associated infection
KAFA	Short Questionnaire for Assessing General and Facet-Specific Job Satisfaction
MDRGN/MRGN	Multidrug-resistant Gram-negative organism (German classification)
MDRO	Multidrug-resistant organism
MRSA	Methicillin-resistant *Staphylococcus aureus*
PPE	Personal protective equipment
SD	Standard deviation
SOP	Standard operating procedure
TeamSTEPPS	Team Strategies and Tools to Enhance Performance and Patient Safety
VRE	Vancomycin-resistant enterococci
WHO	World Health Organization
