# Supplementary material for: Hygiene Knowledge and Digital Health Literacy in Emergency Medical Services in Germany: A Cross-Sectional Study of EMS Personnel and Emergency Physicians with Relevance for the Development of Training Programmes
Source: Healthcare (Basel). 2026 Sep 19;14(18):3091. doi: 10.3390/healthcare14183091 (PMC13606262; doi:10.3390/healthcare14183091)
Supplement: Supplementary file 1 [file healthcare-14-03091-s001.zip › healthcare-4556806-supplementary.pdf]

Supplementary Materials, Table S1: DHLI dimensions by occupational group: proportions of high, medium, and low digital health literacy (n = 72)

**Digital health literacy occupational group**																					
	Navigation skills			Operational skills			Information searching			Contributing own content			Assessing reliability			Determining relevance			Protecting privacy		
	High % (*n*)	Medium % (*n*)	Low % (*n*)	High % (*n*)	Medium % (*n*)	Low % (*n*)	High % (*n*)	Medium % (*n*)	Low % (*n*)	High % (*n*)	Medium % (*n*)	Low % (*n*)	High % (*n*)	Medium % (*n*)	Low % (*n*)	High % (*n*)	Medium % (*n*)	Low % (*n*)	High % (*n*)	Medium % (*n*)	Low % (*n*)
EMS personnel	92.3<br>48	7.7<br>4	0<br>0	98.1<br>51	1.9<br>1	0<br>0	73.1<br>38	25.0<br>13	1.9<br>1	84.6<br>44	11.5<br>6	3.8<br>2	51.9<br>27	38.5<br>20	9.6<br>5	69.2<br>36	26.9<br>14	3.8<br>2	71.2<br>37	23.1<br>12	5.8<br>3
Emergency physicians	65.0<br>13	65.0<br>7	0<br>0	100.0<br>20	0<br>0	0<br>0	55.0<br>11	40.0<br>8	5.0<br>1	70.0<br>14	20.0<br>4	10.0<br>2	25.0<br>5	45.0<br>9	30.0<br>6	60.0<br>12	15.0<br>3	25.0<br>5	55.0<br>11	20.0<br>4	25.0<br>5
Total	84.7<br>61	15.3<br>11	0<br>0	98.6<br>71	1.4<br>1	0<br>0	68.1<br>49	29.2<br>21	2.8<br>2	80.6<br>58	13.9<br>10	5.6<br>4	44.4<br>32	40.3<br>29	15.3<br>11	66.7<br>48	23.6<br>17	9.7<br>7	66.7<br>48	22.2<br>16	11.1<br>8
Note: Classification into high, medium, and low digital health literacy was based on the total score for each DHLI dimension: high = 9–12, medium = 7–8, low = 3–6.																					
